# Case report: Amphiphysin-IgG autoimmunity: a paraneoplastic presentation of appendiceal goblet cell carcinoma

**DOI:** 10.3389/fimmu.2022.1001264

**Published:** 2023-01-04

**Authors:** Jingfang Lin, Tianping Yu, Minjin Wang, Jierui Wang, Jinmei Li

**Affiliations:** ^1^ The department of Neurology, West China Hospital, Sichuan University, Chengdu, Sichuan, China; ^2^ The department of Pathology, West China Hospital, Sichuan University, Chengdu, Sichuan, China; ^3^ Department of Laboratory Medicine, West China Hospital of Sichuan University, Chengdu, Sichuan, China

**Keywords:** amphiphysin, appendiceal goblet cell carcinoma, paraneoplastic neurological syndromes, tremors, pathological findings

## Abstract

**Background:**

Appendiceal goblet cell carcinoma (aGCC) is a rare neoplasm with mixed endocrine and exocrine features. No paraneoplastic neurological syndromes or autoantibodies have been identified in cases of aGCC or even appendiceal tumors. Amphiphysin-immunoglobulin G (IgG) autoimmunity was first described in stiff-person syndrome with breast cancer. We firstly described the clinical course and pathological findings of a patient with aGCC-associated amphiphysin-IgG autoimmunity.

**Case presentation:**

A 54-year-old man who developed aGCC was admitted for acute disturbance of consciousness, psychiatric symptoms, cognitive impairment, seizure and hypotension. Amphiphysin-IgG was detected in the patient’s serum and CSF by immunoblotting and tissue-based indirect immunofluorescence assay confirming the diagnosis of definite paraneoplastic amphiphysin-IgG-positive encephalitis. Histopathology revealed amphiphysin protein expression and accompanying immune cell infiltration (predominantly CD20+ B cells, CD3+ and CD8+ T cells) within the tumor tissue, suggesting a possible paraneoplastic origin of amphiphysin-associated paraneoplastic neurological syndromes (PNSs) in this case. Although the patient’s symptoms resolved after high-dose corticosteroid therapy, he experienced recurrence 6 months later, manifesting as paraneoplastic cerebellar dysfunction. Despite treatment with IV cyclophosphamide and oral mycophenolate mofetil, no improvement was noted.

**Conclusions:**

This case suggests that aGCC may trigger amphiphysin-IgG autoimmunity.

## Introduction

Amphiphysin-IgG autoimmunity was reported in 1992 by Lichte et al. The clinical presentations associated with amphiphysin antibodies (Abs) include stiff-person syndrome, limbic encephalitis (LE), paraneoplastic cerebellar dysfunction (PCD), myelopathy, and peripheral neuropathies (1).

Malignancies such as small cell lung cancer and breast cancer triggered amphiphysin-Abs associated paraneoplastic neurological syndromes (PNSs) have been reported (1). Appendiceal goblet cell carcinoma (aGCC), recognized by the 2019 World Health Organization classification of digestive system tumors, is a rare neoplasm with mixed endocrine and exocrine features (2). Here, we first report a patient with aGCC who developed LE related to amphiphysin-Abs after tumor removal. Although the patient’s symptoms resolved after immunotherapy, he experienced recurrence of PCD. Histopathology revealed amphiphysin protein expression and accompanying immune cell infiltration within the tumor tissue, suggesting a possible paraneoplastic origin of amphiphysin-associated PNSs in this case.

## Case presentation

A 54-year-old male presented with disturbance of consciousness, psychiatric symptoms and behavioral change, cognitive impairment, seizure and hypotension for 11 days. His family history was unremarkable. Exposure to toxic substances was denied. The day before his first symptom, a resection of his right colon was performed in a local hospital, and aGCC was diagnosed by the pathologist.

**Figure 1:**
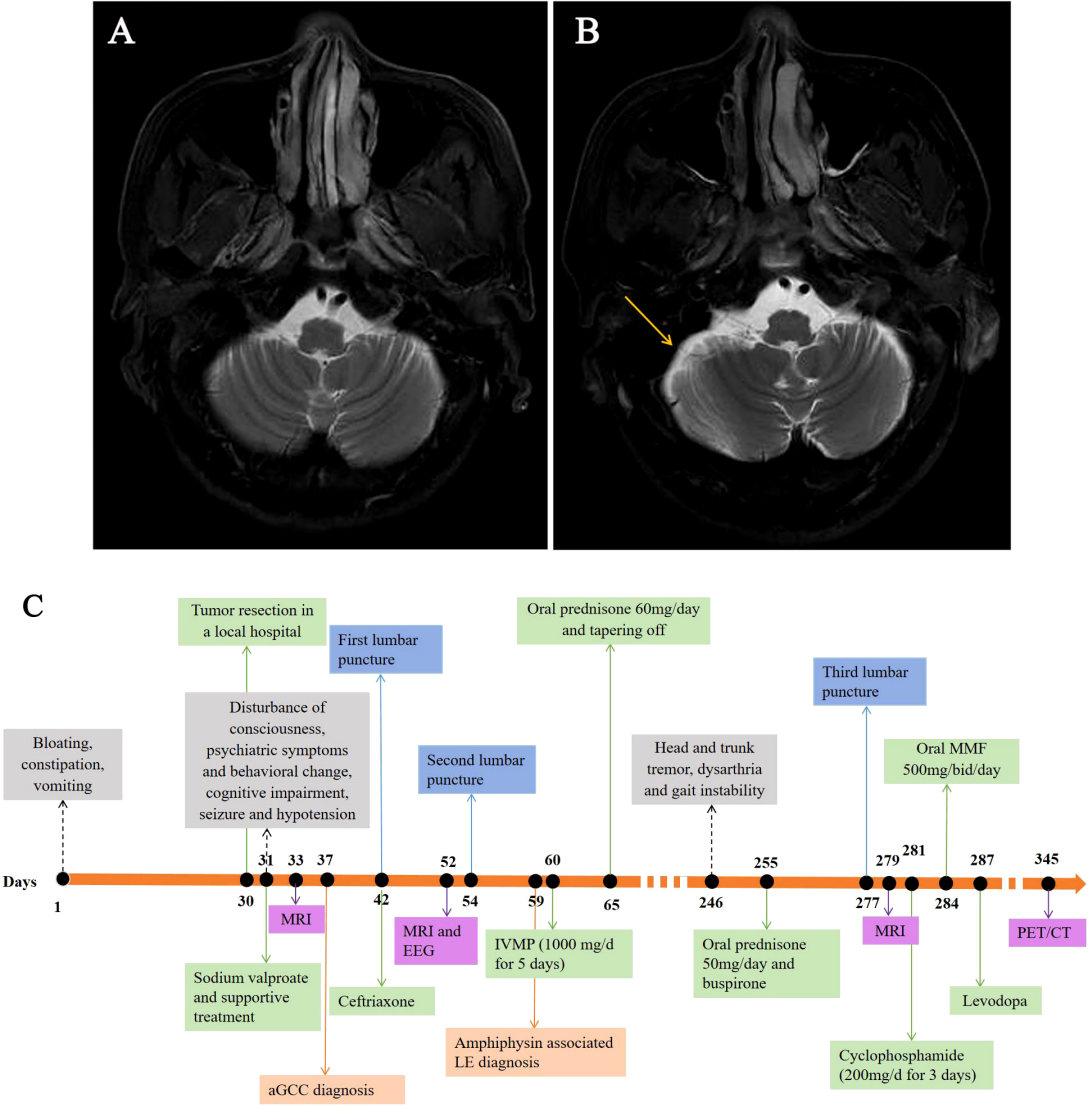
Brain MRI scans obtained from the patient and timeline of his clinical course. Despite there being no specific findings upon an initial T2-weighted MRI **(A)**, mild cerebellar atrophy was observed in the subsequent axial T2-weighted MRI performed seven months later (**B**, yellow arrow). Timeline of the patient’s clinical course highlighting the manifestation, auxiliary examination, treatment and diagnosis time **(C)**. EEG, electroencephalograph; LE, limbic encephalitis; MMF, mycophenolate mofetil; MRI, magnetic resonance imaging; IVMP, intravenous methylprednisolone; PET/CT, positron emission tomography with computed tomography.

Brain magnetic resonance imaging (MRI; **Figure 1A**), routine cerebral spinal fluid (CSF) analyses (**Supplementary file**), and routine laboratory tests were unremarkable (**Table 1**), except for increased carbohydrate antigen 125 (35.2 U/ml; normal range, < 24 U/ml) and blood ammonia (53.7 µmol/L; normal range, 9-33 µmol/L). The anti-amphiphysin antibody tested by immunoblotting (EUROIMMUN, Lübeck, Germany) was positive in both CSF and serum. The diagnosis of amphiphysin-positive LE associated with aGCC was made. A high dose of methylprednisolone (1000 mg/d for 5 days) was administered, followed by a tapered course of prednisone. The symptoms of psychiatric symptoms and seizures then disappeared one month later, but the patient remained cognitive dysfunction (Mini-mental State Examination score = 9).

**Table 1:** Results of additional auxiliary examinations.

Cerebrospinal fluid (the second spinal tap)	
Protein	Normal
Pleocytosis	Normal
Intrathecal IgG synthesis	Normal
Glucose	Normal
Chlorine	Normal
Malignant cell	Normal
Flow cytometry	Normal
Acid-fast bacilli cultures	Normal
Fungus (smear/culture)	Normal
Bacteria (smear/culture)	Normal
IgG Index	Normal
^a^Neuronal cell surface antibodies	(CSF/serum)
Anti-N-methyl-D-aspartate receptor	(Negative/Negative)
Anti-gamma-aminobutyric acid-B receptor	(Negative/Negative)
Anti-leucine-rich glioma-inactivated protein 1 receptor	(Negative/Negative)
Anti-contactin-associated protein-like 2 receptor	(Negative/Negative)
Anti-AMPAR	(Negative/Negative)
Anti-metabotropic glutamate receptor 5 receptor	(Negative/Negative)
Anti-dipeptidyl peptidase-like protein-6 receptor	(Negative/Negative)
Anti-Dopamine-2 receptor	(Negative/Negative)
Anti-IgLON5	(Negative/Negative)
Anti-alpha-amino-3-hydroxy-5-methyl-4-iso-xazolepropionic acid receptors	(Negative/Negative)
^b^Onconeuronal antibodies	(CSF/serum)
Anti-Hu	(Negative/Negative)
Anti-Ri	(Negative/Negative)
Anti-Yo	(Negative/Negative)
Anti- Amphiphysin	(Positive/Positive)
Anti-GAD	(Negative/Negative)
Anti-PCA2	(Negative/Negative)
Anti-Ma1	(Negative/Negative)
Anti-Ma2	(Negative/Negative)
Anti-CV2/CRMP5	(Negative/Negative)
Anti-SOX1	(Negative/Negative)
Blood ammonia	Abnormal (53.7 umol/L; normal range: 9-33 umol/L)
Aspartate aminotransferase	Normal
Alanine aminotransferase	Normal
Vitamin B12	Normal
Intrinsic Factor	Normal
C-reactive protein	Abnormal (19.8 mg/L; normal range: < 5 mg/L)
Procalcitonin	Abnormal (0.2 ng/ml; normal range: < 0.046 ng/ml)
Microbiological exams	(Serum)
Human immunodeficiency virus	Normal
Syphilis	Normal
Mycobacteria	Normal
Human cytomegalovirus	Normal
Epstein-Barr virus	Normal
Parasite IgG antibody	Normal
Tumour markers	(Serum)
Carbohydrate antigen 125	Abnormal (35.2 U/ml; normal range: < 24U/ml)
Carbohydrate antigen 19-9	Normal
Carbohydrate antigen 72-4	Normal
Carbohydrate antigen 15-3	Normal
Carcinoembryonic antigen	Normal
Alpha fetoprotein	Normal
Prostate-specific antigen	Normal
*Thyroid function	(Serum)
Triiodothyronine	Abnormal (0.82 nmol/L; normal range: 1.3-3.1 nmol/L)
Free triiodothyronine	Abnormal (2.6 pmol/L; normal range: 3.6-7.5 pmol/L)
Free thyroxine	Abnormal (11.5 pmol/L; normal range: 12.0-22.0 pmol/L)
Thyroxine	Abnormal (57.8 pmol/L; normal range: 62-164 pmol/L)
Thyroid-stimulating hormone	Normal
Thyroid globulin antibody	Normal
Thyroid peroxidase antibody	Normal
Triiodothyronine	Normal
Cortisol testing	Normal
Immunological test results	(Serum)
Anti-nuclear antibody	Normal
Anti-keratin antibodies	Normal
Anti-double-stranded deoxyribonucleic acid	Normal
Anti-Smith antibodies	Normal
Anti-SS-A and SS-B	Normal
Anti-Neutrophil Cytoplasmic antibodies	Normal
Anticardiolipin antibodies	Normal
Rheumatoid factor	Normal
Electroencephalogram	Predominantly frontotemporal slow activity
Electromyogram	Normal
Chest-abdominal CT	Normal
Abdomen CT	Normal
Brain computed tomography angiography	Normal
Spine magnetic resonance imaging	Normal
Positron emission tomography with computed tomography scans	Normal

AMPAR, α-amino-3-hydroxy-5-methyl-4isoxazolepropionic acid receptor; Anti-SS-A and SS-B, Anti-SS-A(Ro) and anti-SS-B(La) autoantibodies; CSF, cerebrospinal fluid.

a = cell base assay and tissue-based indirect immunofluorescence assay (EUROIMMUN, Germany).

b = indirect immunofluorescence and tissue-based indirect immunofluorescence assay (EUROIMMUN, Germany).

* Repeat thyroid function test after 1 month was normal.

Unfortunately, seven months later, the patient was readmitted to the hospital with progressive head and trunk tremors, dysarthria and gait instability for over 1 month. Neurological examination revealed head and trunk tremors, cognitive dysfunctions, bilateral gaze-evoked nystagmus and cerebellar ataxia (Vedio1). CSF analysis remained unchanged. Brain MRI showed mild cerebellar atrophy (**Figure 1B**). Amphiphysin-IgG was still present in CSF and serum. Positron emission tomography with computed tomography (PET/CT) scans revealed no tumor metastasis. The patient received IV cyclophosphamide (200 mg/d) and oral mycophenolate mofetil (500 mg/d) therapy. There was no improvement or deterioration of the neurological symptoms 3 months later. The evaluation of PNSs process is summarized in **Figure 1C**.

**Figure 2:**
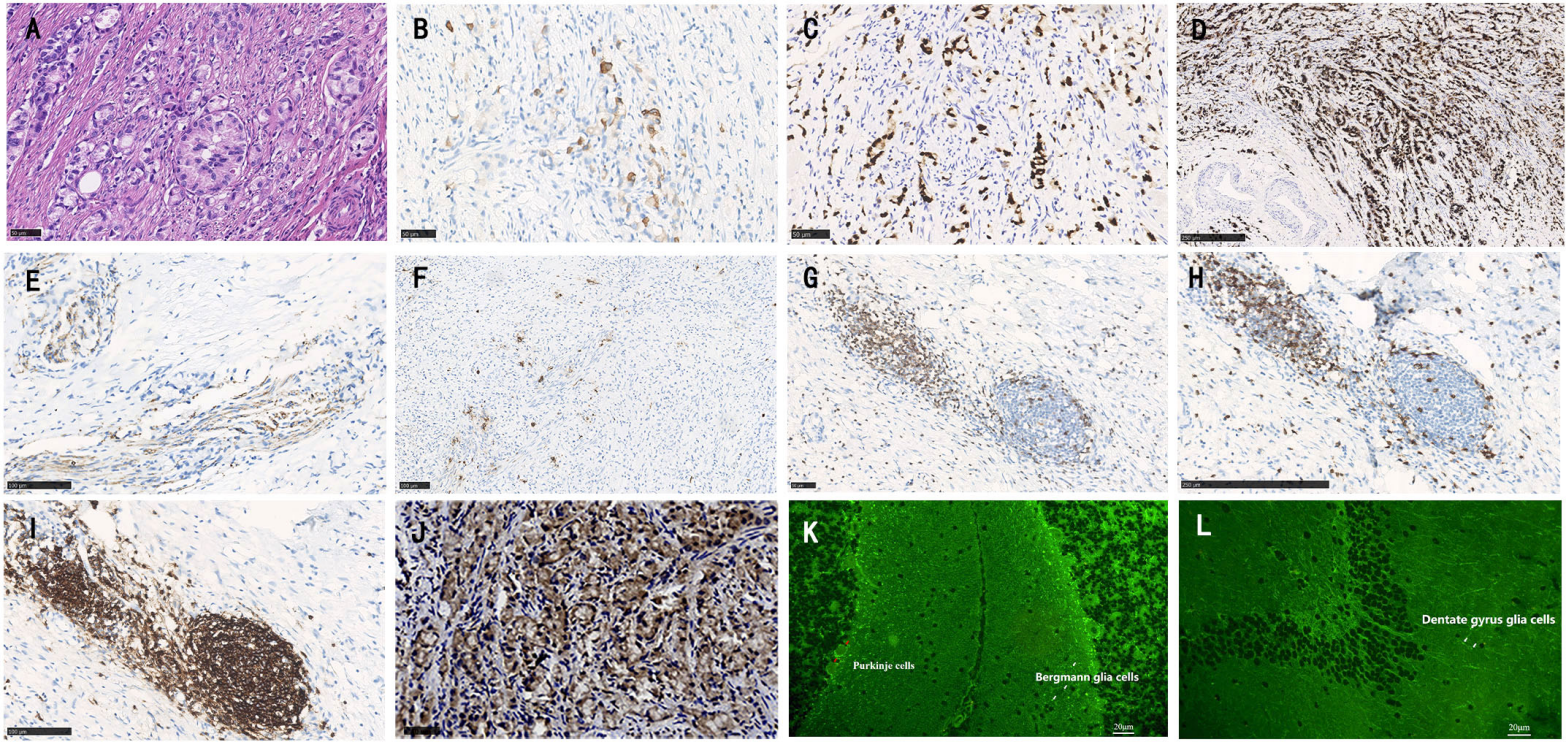
Histopathology of appendiceal goblet cell carcinoma of the patient. Tumor cells exhibit a goblet cell morphology and characteristically form small tight clusters **(A)**. The immunohistochemical staining shows moderate positivity for CK20 **(B)**, CDX-2 **(C)**, and strong positivity for carcinoembryonic antigen (CEA, D). Staining for synaptophysin (Syn, E), and chromogranin **(F)** is mild. Varying degrees of positivity are also shown in lymphocytes such as CD3+ T cells **(G)**, CD8+ T cells **(H)** and CD20+ B cells **(I)**. Immunohistochemistry shows positive staining for amphiphysin-IgG **(J)**. Tissue-based indirect immunofluorescence assay (TBA) of the patient’s serum detected fluorescence responses, with specific fluorescence responses in Purkinje cells (red arrows) from the monkey cerebellum brain section **(K)** as well as dentate gyrus glial cells (white arrows) from the monkey hippocampal tissue. **(A,–C, G, J)** bar = 50 µm; D, H bar = 250 µm; **(E, F, O)** bar = 100 µm, **(K, L)** bar = 20 µm).

To investigate a possible paraneoplastic origin of amphiphysin-PNSs in this patient, tissue blocks of the aGCC were examined pathologically. Immunohistochemical staining for CK20, CDX-2, carcinoembryonic antigen (CEA) and neuroendocrine markers (synaptophysin and chromogranin) were positive (**Figures 2A–F**), which meets the diagnostic pathological diagnostic criteria of Agcc according to the 2019 WHO.^2^ The expression of Ki67 protein was about 10%. MUC1 and MUC2 were also positive. Immunohistochemistry revealed positive CD3+ T cells, CD8+ T cells and CD20+ B cells in the tumor tissue (**Figures 2G–I**). However, CK7, CD138+ plasma cells, CD68+ macrophages, and IgG were negative in the tissue. Tumor cells showed reactivity with amphiphysin-Abs (**Figure 2H**), which indicates that the aGCC might play a role in triggering amphiphysin-IgG autoimmunity. Tissue-based indirect immunofluorescence assay (TBA) of the patient’s serum revealed IgG antibody to Purkinje cells of the monkey cerebellum and dentate gyrus glia cells of the monkey hippocampus (**Figures 2K, L**).

## Diagnostic assessment

According to the clinical manifestations, tumor, positive anti-amphiphysin antibody in CSF and serum, and well response to immunotherapy, aGCC associated amphiphysin-IgG autoimmunity was considered in this patient.

## Discussion

This case presents several novelties. This is the first case report of aGCC associated PNSs to date. Although the etiology of PNSs remains unknown, it is proposed that the expression of neuronal antigens within tumors could initiate an autoimmune response that leads to the production of autoantibodies (3). Immunopathological analysis of amphiphysin-IgG autoimmunity may help to elucidate the underlying pathophysiological mechanism of the disease, but unfortunately, published data are scarce, to date, brain and/or spinal cord immunopathological reactions have only been studied in 4 patients (4–7). In those cases, biopsies revealed marked inflammatory responses in perivascular and parenchymal regions. Both T (especially CD3+ and CD8+ T cells) and B lymphocytes (especially CD20+ B cells) were present in the lesions, and CD68+ microglial cells were abundant in the pons in one previous patient. In addition, only 14 cases reported pathological findings coming from concomitant neoplasms (small cell lung cancer, breast cancer and non-small cell lung cancer) in which amphiphysin-IgG was present in patients’ tumor tissues, which correlated with the patients’ antibody specificity (8, 9). In this case, immunohistochemical amphiphysin-Abs positivity was shown in appendiceal tumor tissue, confirming that the syndrome was triggered by the tumor.

In addition, in the literature, most of autoimmune cerebellar dysfunction is characterized by limb or trunk tremors, and only a few cases of autoimmune cerebellar dysfunction associated head tremor have been reported (10–12). Head tremor had not been reported in amphiphysin-Abs associated PNSs. From the largest series reported by Pittock et al., a minority of patients with amphiphysin-IgG presented with involuntary movement, including mandibular involuntary movement, chorea and truncal tremor (4). In this case, considering the progressive cerebellar atrophy shown by the brain MRI, tremors in this patient were considered of cerebellar origin.

It is unusual that neurological symptoms occur shortly after tumor resection, which might be due to the immune trigger from antigen exposure at the time of resection (13). In this case, the onset of PCD seven months after tumor resection is also extremely rare. Since whole body PET/CT was negative, and the patient is still stable on regular follow-up after the occurrence of cerebellar dysfunction, the evidence of tumor recurrence or metastasis is insufficient. To the best of our knowledge, there are only a few published cases describe the recurrence of paraneoplastic neurologic syndromes after tumor resection, but without evidence of tumor recurrence (14–16). However, owing to their rarity, the mechanism of this condition is still unknown. Moreover, the case showed a different phenotype and treatment response during disease onset compared to relapse, which is unusual in previously reported autoimmune encephalitis (17). He improved during the first stage of the disease when limbic encephalitis was predominant. Nevertheless, during relapse characterized by cerebellar dysfunction despite aggressive treatment regarding second-line immunotherapy administration, no improvement was noted. Only stabilization of the clinical symptoms was achieved. We suggested for the patient to receive intravenous immunoglobulin and other immunotherapy. Unfortunately, due to economic reasons, the patient refused.

In conclusion, this case suggests that aGCC may trigger amphiphysin-IgG autoimmunity, and amphiphysin-IgG autoimmunity could relapse with a different clinical phenotype months after the initial episode, so long-term follow-up is needed.

## Data availability statement

The original contributions presented in the study are included in the article/**Supplementary Material**. Further inquiries can be directed to the corresponding author.

## Ethics statement

Written informed consent was obtained from the individual(s) for the publication of any potentially identifiable images or data included in this article.

## Author contributions

JFL and TY drafted and revised the manuscript. MW and JW collected and interpreted the data. JML designed the study and revised the manuscript. All authors contributed to the article and approved the submitted version.

## Supplementary material

Supplementary Video 1 Neurological examination revealed head and trunk tremors, bilateral gaze-evoked nystagmus and cerebellar ataxia.
