# Supplementary material for: Case report: Amphiphysin-IgG autoimmunity: a paraneoplastic presentation of appendiceal goblet cell carcinoma
Source: Front Immunol. 2023 Jan 4;13:1001264. doi: 10.3389/fimmu.2022.1001264 (PMC9845691; doi:10.3389/fimmu.2022.1001264)
Supplement: Supplementary file 1 [file Table_1.pdf]

eTable 1. Results of three spinal taps

Cerebrospinal fluid	First spinal tap	Second spinal tap	Third spinal tap
Protein	Normal	Normal	Normal
Pleocytosis	Normal	Normal	Normal
Intrathecal IgG synthesis	NA	Normal	Normal
Glucose	Abnormal (132 mmol/L; normal range: 120-130 umol/L)	Normal	Normal
Chlorine	Normal	Normal	Normal
Malignant cell	Normal	Normal	Normal
Flow cytometry	NA	Normal	Normal
Acid-fast bacilli cultures	Normal	Normal	Normal
Fungus (smear/culture)	Normal	Normal	Normal
Bacteria (smear/culture)	Normal	Normal	Normal
IgG Index	NA	Normal	Normal

NA: not available.
